# Solid Lipid Nanoparticles of Atovaquone Based on 2^4^ Full-Factorial Design

**Published:** 2015

**Authors:** Noratiqah Mohtar, Nurzalina A. K. Khan, Yusrida Darwis

**Affiliations:** School of Pharmaceutical Sciences, Universiti Sains Malaysia, 11800 Pulau Pinang, Malaysia.

**Keywords:** Solid lipid nanoparticles, Factorial design, Atovaquone, Triglycerides, Soy lecithin

## Abstract

Solid lipid nanoparticles of atovaquone (ATQ-SLN) were prepared by high shear homogenization method using tripalmitin, trilaurin, and Compritol 888 ATO as the lipid matrices and Phospholipon 90H, Tween 80, and poloxamer 188 as the surfactants. Optimization of the formulations was conducted using 6 sets of 2^4^ full-factorial design based on four independent variables that were the number of homogenizing cycles, concentration of the lipid, concentration of the co-surfactant, and concentration of the main surfactant. The dependent variables were particle size and polydispersity index (PdI). The homogenizing cycles showed a negative influence on the dependent variables which reduced both the particle size and the PdI value. Moreover, a combination of certain percentages of the main surfactant and co-surfactant also showed a negative influence that reduced both the particle size and PdI value. Selected formulations from each design were further characterized for the entrapment efficiency and yield. The optimised formulation of ATQ-SLN consisted of trilaurin, Phospholipon 90H and Tween 80 with a particle size of 89.4 ± 0.2 nm and entrapment efficiency of 83.0 ± 1.7%. The *in-vitro* release evaluation of the formulation showed a complete and immediate release of ATQ from the SLN that could be a solution to improve the poor aqueous solubility and hence poor bioavailability of the drug.

## Introduction

Atovaquone (ATQ) or *trans*-2-[4-(4-chlorophenyl) cyclohexyl]-3-hydroxy-1, 4-naphthalenedione is an agent with antiparasitic characteristics for the treatment of pneumocystis pneumonia (1, 2), malaria (3) and toxoplasmosis (4). It is a structural analogue of protozoan ubiquinone (also known as coenzyme Q) that targets the mitochondrion by inhibiting the electron transport and causes the collapse of the membrane potential in such organelles (5). ATQ is practically insoluble in water and belongs to class II in the bio pharmaceutics classification system (BCS) which exhibits a low aqueous solubility due to the hydrophobic nature but high membrane permeability. Dissolution of the drug in the intestinal medium is the rate-limiting step of absorption and any modification to improve the solubility and dissolution rate in the luminal tract will enhance the absorption (6). Food has been reported by Rolan *et al*. (1994) to be able to increase the absorption of ATQ when administered in the tablet form. The area under the curve (AUC) and maximum concentration (C_max_) were increased by 3.3-fold and 5.3-fold, respectively, in the administration of ATQ tablet after a high-fat breakfast when compared with administration under fasting conditions (7).

ATQ has been previously incorporated into several types of nano formulations including nanocapsules, nanosuspension and liposomes (8-10). A solid lipid nanoparticle formulation offers several advantages including the ability to increase lipophilic drug bioavailability and controlled-release delivery (11, 12). The formulation can be a solution to avoid bio toxicity problems as it uses physiological lipids such as triglycerides and natural surfactants such as soy lecithin which are generally recognized as safe (GRAS) (13).

Conventional pharmaceutical development approach involves changing one parameter at a time during the optimization of formulation variables. Nonetheless, this approach could not define the impact and interaction between variables. Hence, factorial design study could be utilized to overcome this issue by enabling simultaneous variation of the factors to quantify the outcomes caused by the independent variables and interaction between the variables. Furthermore, several researchers have implemented factorial design as part of the development process of nano formulations (14, 15). 

The aim of the present study was to develop an optimized formulation of ATQ-loaded SLN using a 2^4 ^full factorial design and to evaluate the dissolution of the formulation in the simulated gastric and intestinal fluid. Factorial design was used to investigate the influence of four independent variables, namely homogenizing cycles, lipid concentration, co-surfactant concentration, and main surfactant concentration, towards two dependent variables, namely particle size and polydispersity index (PdI). Two types of triglycerides, tripalmitin (C16) and trilaurin (C12), were used to study the influence of chain length while Compritol ATO 888 (a mixture of mono-, di- and triglycerides) was used to study the influence of the lipid arrangement on the characteristics of the produced SLN. Two non-ionic surfactants (Poloxamer 188 and Tween 80) were used as co-surfactants while a hydrogenated soy lecithin (Phospholipon 90H) was used as a main surfactant. The best formulation in the factorial design was further optimized and evaluated in the *in-vitro* release study.

## Experimental

*Materials*

Atovaquone was purchased from Hallochem Pharmaceutical Co. Ltd. (Chongqing, China) and tripalmitin was obtained from International Laboratory (USA). Trilaurin was purchased from Chemos GmbH (Regenstauf, Germany) and Compritol ATO 888 was a kind gift from Gattefossè (USA). Phospholipon 90H was obtained from Lipoid (Steinhausen, Germany) and Poloxamer 188 was purchased from Molekula Ltd. (Dorset, UK). Tween 80 was obtained from R&M Chemicals (Essex, UK) and acetonitrile (HPLC grade) was bought from J. T. Baker (Phillipsburg, USA).

*Preparation of ATQ-SLN*

The lipid was melted at 5-10°C above the melting point in a water bath. ATQ was incorporated into the lipid melt prior to dispersion of Phospholipon 90H in the mixture. Simultaneously, an aqueous phase was separately prepared at the same temperature by dissolving non-ionic surfactants in distilled water so that the end weight was 20 g. The pre heated aqueous phase was added into the lipid phase and subjected to high shear homogenization by using the ULTRA TURRAX^®^ T25 Basic (IKA, USA) at 20,000 rpm for different homogenizing cycles (5 min for each cycle). Finally, the preparation was left to cool to 23-25°C before further characterization.

*2* ^4^ *full-factorial designs in three replicates*

Homogenizing cycles and composition of each material were optimized using 2^4^ full-factorial designs by using four independent variables with 2 levels for each variable. The lower and higher values of each factor are presented in Table 1. The factorial design experiments were carried out in six sets of different combinations of lipids and surfactants as shown in Table 2. The evaluated responses were the average particle size (Zave) and polydispersity index (PdI). The experiment was done in triplicate and all results were analyzed statistically using Design-Expert^®^ Software 6.0.10 (Stat-Ease, Inc, USA). All preparations were randomly prepared as designed by the software and the significance of interaction between variables was evaluated using ANOVA. In the analysis, important effects were chosen from a half probability plot to be included in the model while remaining effects were excluded as residuals. Data transformation was made where necessary in order to be analyzed using ANOVA and the coefficient of every significant effect was further used to develop a reduced equation using multiple regression analysis. Some of the interactions between independent variables were visually explained by using 3D surface plots. From all sets of designs, formulations with particle sizes < 500 nm and PdI ≤ 0.5 were chosen to further characterize the entrapment efficiency (% EE) and yield (% Yield).

**Table 1:** Independent variables with high and low levels

**Independent variables**	**Low level**	**High level**
Factor A (Homogenizing cycles)	2 (-)	6 (+)
Factor B (Lipid concentration)	0.5 %w/w (-)	1.5 %w/w (+)
Factor C (Co-surfactant concentration)	0.25 %w/w (-)	0.75 %w/w (+)
Factor D (Main surfactant concentration)	0.25 %w/w (-)	0.75 %w/w (+)

**Table 2:** List of lipids and surfactants for each factorial design

**Factorial design**	**Lipid**	**Main surfactant**	**Co-surfactant**
1	Tripalmitin	Phospholipon 90H	Poloxamer 188
2	Tripalmitin	Phospholipon 90H	Tween 80
3	Trilaurin	Phospholipon 90H	Poloxamer 188
4	Trilaurin	Phospholipon 90H	Tween 80
5	Compritol 888 ATO	Phospholipon 90H	Poloxamer 188
6	Compritol 888 ATO	Phospholipon 90H	Tween 80

*Optimization of the incorporated ATQ*

The final formulation was selected for further characterization using different amounts of ATQ. Different amounts of ATQ were incorporated into the lipid melt in the initial stage of the SLN preparation and the SLN was evaluated for particle size, PdI, entrapment efficiency and yield.

*Particle size analysis*

Photon correlation spectroscopy (PCS) was used for the determination of particle size (Zave) and polydispersity index (PdI) of all formulations. The measurements were done using Zeta sizer 1000 HS_A_ (Malvern Instruments, UK) at a wavelength of 633 nm and a fixed angle of 90°. The scattering intensity of the dispersion was adjusted to be within 100 to 150 kilo counts per second (Kcps) by diluting the samples with 0.45 µm membrane filtered distilled water. Each dispersion was measured in triplicate at 25°C for a total duration of 120 s and 10 s delay between measurements.

*Determination of entrapment efficiency, yield and drug loading*

The entrapment efficiency (%EE) was determined by separating the free drug from the SLN using gel filtration chromatography. In the separation, 1 ml of the SLN was run through a Sephadex G25 (Sigma-Aldrich, U.S.A) column and the opalescent eluent was further collected and freezed at -80°C before lyophilization. The drying was conducted for 24 hours in the freeze drying system (Labconco 753501, USA) equipped with a condenser operating at -50°C. The lyophilized sample was dissolved in chloroform to break the nanoparticles prior to evaporation under nitrogen blow at 40°C. The dried layer was reconstituted using acetonitrile and 20 µl of the sample was injected into the HPLC for analysis. The quantified amount of ATQ was considered as the encapsulated ATQ and the entrapped drug was calculated according to Equation 1. The yield (%Yield) and drug loading (%DL) were calculated according to Equation 2 and Equation 3, respectively. 

$\mathrm{\%EE}=\frac{\mathrm{Weight of encapsulated ATQ}}{\mathrm{Initial weight of ATQ and excipients}}\times 100\%$

(Eq. 1)

$\mathrm{\%Yield}=\frac{\mathrm{Weight of recoverd SLN}}{\mathrm{Initial weight of ATQ and excipients}}\times 100\%$

(Eq. 2)

$\mathrm{\%DL}=\frac{\mathrm{Weight of encapsulated ATQ}}{\mathrm{Weight of recoverd SLN}}\times 100\%$

(Eq. 3)

*HPLC analysis*

The analysis was performed using a C_18_ column (Phenomenex, 150 x 4.60 mm ID, 5 µm) fitted with a universal guard column (Thermoscientific, 4 x 4.6 mm ID) at 45°C. The separation was run using 0.02 M ammonium acetate pH 3 (adjusted with glacial acetic acid) and acetonitrile at a ratio of 15:85 (v/v) as the mobile phase. The volume of injection was 20 µl. The detection wavelength was set at 254 nm and the flow rate was maintained at 1 ml/min.

The HPLC method was validated according to USFDA guidelines (16) for linearity, limit of detection (LOD), limit of quantification (LOQ), intra-day and inter-day validation. Precision (%RSD = % relative standard deviation) and accuracy (%RE = % relative error) of all validated parameters were calculated according to Equation 4 and Equation 5 respectively.

(Eq. 4) $$\mathrm{\%RSD}=\frac{\mathrm{Standard deviation}}{\mathrm{Mean value}}\times 100\%$$

$\mathrm{\%RE}=\frac{\left(\mathrm{Calculated concentration}-C_{\mathrm{std}}\right)}{C_{\mathrm{std}}}\times 100\%$

(Eq. 5)

C_std_ is the nominal concentration of the standard solution (ng/ml).

*Differential scanning calorimetry*

The thermal analysis was carried out by accurately weighing 5-7 mg of the sample in an aluminium pan (Perkin-Elmer, UK) and the pan was sealed non-hermetically. The DSC was performed using a Perkin-Elmer Pyris 6 DSC (Beaconsfield, UK) equipped with an intracooler at 10°C/min. Helium was used as the purge gas at a rate of 20 ml/min. The sample was scanned in triplicate and the melting point was determined as the temperature at the onset of the endothermic drop.

*Transmission electron microscopy (TEM)*

The shape of the SLN in suspension was examined using a TEM. A small drop of the sample was applied to a copper grid and left for 15 minutes. Then the excess fluid was removed using filter paper and left to dry prior to the examination under the TEM (CM12, FEI, Eindhoven, The Netherlands).

*In-vitro release study*

The *in-vitro* release study of ATQ from the SLN formulation was performed in a simulated gastric fluid (SGF) at pH 1.2 and a simulated intestinal fluid (SIF) at pH 6.8, both without enzymes according to USP. Poloxamer 188 (1%w/w) was added into the release medium to improve wetting of the drug and nanoparticles. In this study, 0.7 ml of ATQ-SLN was dispersed into 150 ml of the release media and the medium was stirred at 100 rpm at 37 ± 2°C. 1 ml of aliquot was drawn at predetermined intervals of 5, 10, 15, 30 and 60 min and filtered using poly tetra fluoro ethylene (PTFE) filters (Titan 2, USA), with a 0.45µm pore size. 20µl of the filtered sample was directly injected into the HPLC and the drawn aliquot was immediately replaced with an equal volume of the fresh dissolution media. The release study was also conducted with an equal amount of pure ATQ in both media as comparison.

## Results

*Factorial design*

**Figure 1:**
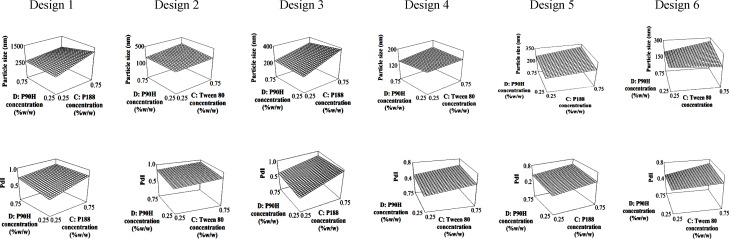
3D response surface plots for factor C and D (P90H: Phospholipon 90H, P188: Poloxamer 188).

A full 2^4^ factorial design was conducted to find the best formulations from six possible combinations of chosen lipids and surfactants. The deduced equations for particle size and PdI are presented in Table 3. The contribution of every independent factor towards the particle size and PdI of the produced SLN in every factorial design was concluded in Table 4. Factors which significantly affected the response parameters (p < 0.05) were labelled with *. From the tabulated result, an increase in the number of homogenizing cycles (factor A) significantly reduced the particle size of the SLN regardless of the type of lipid and surfactant system, while the opposite effect can be seen with the concentration of the co-surfactant (factor C). On the other hand, both co-surfactants and main surfactants (factors C and D) gave a positive impact while the lipid concentration (factor B) gave a negative impact on the PdI. In spite of the individual effects of the co- and main surfactants, interaction between them was favourable in producing an SLN with low particle size and PdI. The 3D response surface plots of the combination of surfactants are depicted in Figure 1 to further explain the interaction between factors in every design.

**Table 3:** Equations for particle size and PdI for every design

**Design**	**Equation for particle size**	**Equation for PdI**
1	**1.0/(Particle size)** = + 2.379E-003 + 6.370E-004A + 1.869E-004B – 8.695E-004C + 1.220E-004D + 7.131E-005AB - 1.239E-004AC + 1.604E-006AD + 1.358E-004BC + 6.434E-005BD + 1.585E-004CD + 7.241E-006ABC + 6.495E-005ABD – 6.454E-005ACD - 8.150E-005BCD - 1.927E-004ABCD	**Polydispersity index** = + 0.72 - 0.017A - 0.048B + 0.11C + 0.050D - 0.047AD - 0.048CD
2	**Log**_10_ ** (Particle size)** = + 2.40 - 0.14A - 7.067E-003B + 0.037C + 0.027D - 0.021AB - 0.051AC + 0.013AD - 0.062BC - 0.012BD + 0.018CD - 0.011ABC + 8.720E-003ABD - 0.031ACD + 0.015BCD	**Polydispersity index** = + 0.84 - 0.100A - 0.070B + 0.049C + 0.051D - 0.052AB - 0.013AC + 0.052AD - 2.021E-003BC + 1.396E-003BD - 1.354E-003CD - 0.017ABC + 0.028ABD - 5.271E-003ACD + 0.015BCD - 0.015ABCD
3	**Particle size** = + 153.18 - 30.14A + 1.05B + 7.21C - 2.11D - 2.92AB - 7.80AC + 1.40AD - 19.21BC - 7.30BD + 5.07CD - 4.67ACD	**1.0/(Polydispersity index**)^1/2^ = + 1.42 + 0.051A + 0.11B - 0.099C - 0.068D + 0.050AB + 0.042AC - 0.022AD + 0.019BC - 0.034BD + 0.019CD + 1.482E-003ABC - 0.040ABD + 0.023ACD + 0.026BCD + 0.027ABCD
4	**1.0/(Particle size)** = + 3.942E-003 + 5.751E-004A - 1.908E-005B - 9.499E-004C - 1.090E-005D + 1.029E-004AB - 3.112E-006AC - 4.147E-005AD + 1.450E-004BC + 2.684E-004BD + 1.951E-004CD - 1.699E-004ABC + 1.350E-004ABD - 1.635E-004BCD	**Polydispersity index** = + 0.73 - 0.011A - 0.040B + 0.18C + 2.104E-003D - 0.030AB + 6.875E-004AC - 4.896E-003BC - 0.052CD + 0.032ABC
5	**1.0/(Particle size)** ^1/2^ = + 0.062 + 1.365E-003A + 3.307E-003B - 2.519E-003C - 2.229E-003D + 1.590E-003AB + 1.041E-003AC - 1.112E-003AD + 1.468E-003BC + 7.609E-004BD - 8.683E-004CD + 5.689E-004ABD - 9.264E-004ACD - 1.364E-003BCD	**Polydispersity index **= + 0.45 + 0.034A - 0.10B + 0.083C + 0.068D - 9.938E-003AB - 9.313E-003AC + 0.026AD - 0.054BC - 0.033BD - 2.188E-003CD - 1.229E-003ABC - 0.012ABD + 0.039ACD - 0.033BCD - 0.026ABCD
6	**(Particle size)** ^-2.2^ = + 9.239E-006 + 1.238E-006A - 1.007E-007B + 2.315E-007C - 1.605E-007D + 1.271E-006AB - 4.438E-007AC - 1.465E-006AD + 8.551E-007BC - 1.217E-006BD - 1.193E-006CD + 6.130E-007ABC - 2.178E-007ABD - 1.090E-006ACD + 4.520E-007BCD + 1.006E-006ABCD	**Polydispersity index** = + 0.60 + 0.029A - 0.12B + 0.068C + 1.389E-003D - 0.040AB + 4.667E-003AC - 0.036AD + 0.015BC + 0.085BD + 0.039CD + 3.056E-003ABC - 0.030ABD + 0.013ACD - 0.014BCD - 0.030ABCD

An optimum combination of surfactants is essential as an increase in particle size and a drop in the quality of the dispersion were recorded when the concentrations of both co-surfactant and Phospholipon 90H were increased simultaneously (Figure 1). The interaction between these two factors can be seen in the response surface plots where formulations with a smaller particle size and lower PdI can be produced using low concentrations of the co-surfactant regardless of the concentration of Phospholipon 90H in all designs.

*Validation of the HPLC method*

The HPLC method was validated according to USFDA (1994) and the retention time of ATQ was found to be at 5.54 ± 0.01 min (*n* = 5). The calibration curve (*n =* 5) was developed in the range of 40 to 4000 ng/ml of ATQ and evaluated for 5 consecutive days. The mean linear regression equation was y = 57.855(± 0.675) x - 198.34(± 41.86) with a coefficient of determination of 0.9999 (± 0.0001).

The limit of detection (LOD) was 0.625 ng/ml while the limit of quantification (LOQ) was found to be at 40 ng/ml with the precision (%RSD) of 2.09% and accuracy (%RE) of 0.06%. The intra-day and inter-day validation were conducted using three concentrations which were 120, 1500 and 3500 ng/ml of ATQ. Each concentration was repeated 5 times in intra-day and 5 times each day for 3 consecutive days in inter-day validation. The intra-day precision ranged between 0.11 to 0.90% while the accuracy ranged between -1.51 to 0.89%. The inter-day precision ranged between 0.54 to 1.24% while the accuracy ranged between -0.09 to 0.41%. The data indicate a good degree of accuracy and precision for the method within and between analytical runs.

*Determination of %EE, %Yield and % DL*

From all six factorial designs, formulations with a particle size of < 500nm and PdI of ≤ 0.5 were selected for further evaluation. The %EE and %Yield of all chosen formulations were tabulated in Table 5. In this study, the utilization of Compritol 888 ATO as a lipid matrix resulted in the production of SLN formulations with the desired particle size and PdI (Design 5 and 6), but lower encapsulation ability. On the other hand, higher encapsulation of ATQ could be seen in the trilaurin-and tripalmitin-SLN system. Among the formulations studied, TLT 16 was selected for further evaluation because of its highest %EE which was 45.7 ± 1.8%. The calculated %DL for TLT 16 was 0.75 ± 0.03% and TLT 16 was formulated using trilaurin, phospholipon 90H and Tween 80. 

*Optimization of the drug loading*

The amount of ATQ incorporated inside the SLN was reduced to optimize the %EE of ATQ in TLT 16. Reducing the amount of ATQ from 10 mg to 3 mg improved the % EE of the formulation from 45.7 ± 1.8 to 83.0 ± 1.7% with a significant reduction (p < 0.05) in particle size of TLT 16 from 95.3 ± 0.9 to 89.4 ± 0.2 nm. 

*Characterization of ATQ-SLN*

**Figure 2:**
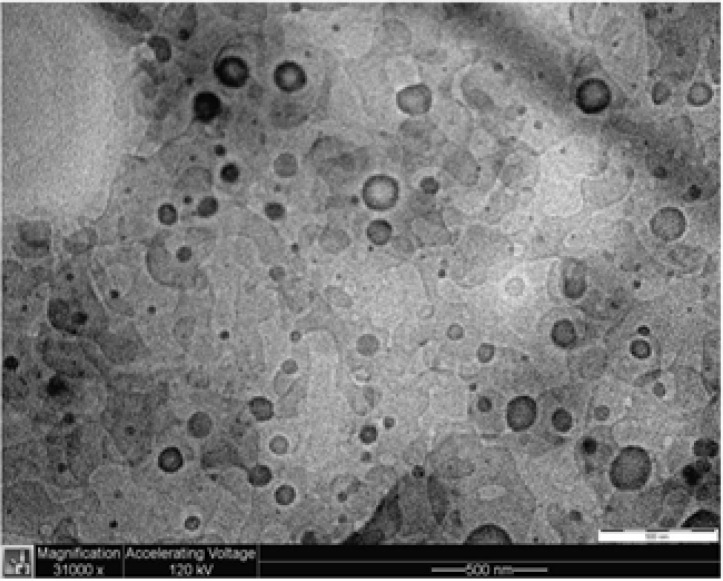
TEM image of TLT 16 suspension under 31,000 magnification

The particle shape and size of the ATQ-SLN in TLT 16 were examined under an electron microscope. Observation of the suspension under a TEM indicated the spherical shape of the SLN with most particles having a size of less than 100 to 200 nm (Figure 2). 

**Figure 3:**
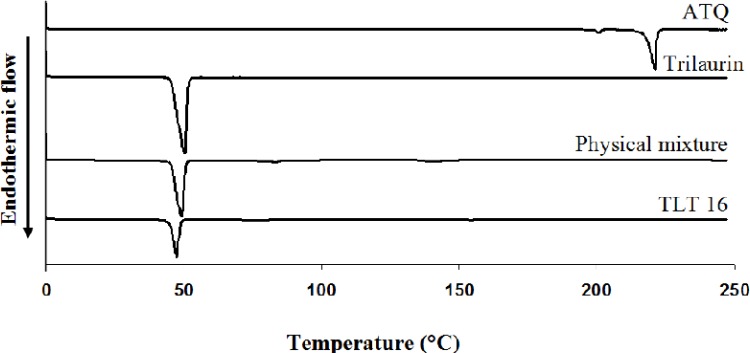
DSC thermograms of ATQ, trilaurin, physical mixture, and ATQ-SLN formulation (TLT 16).

In the thermogram (Figure 3), the melting temperature of pure trilaurin was 45.77 ± 0.03^o^C and the temperature was slightly shifted to the right to 45.99 ± 0.01^o^C in the physical mixture. On the other hand, the melting point was significantly shifted to the left to 44.65 ± 0.34^o^C in ATQ-SLN (TLT 16) indicating physical interactions between lipid and the excipients.

*In-vitro release study*

**Figure 4.**
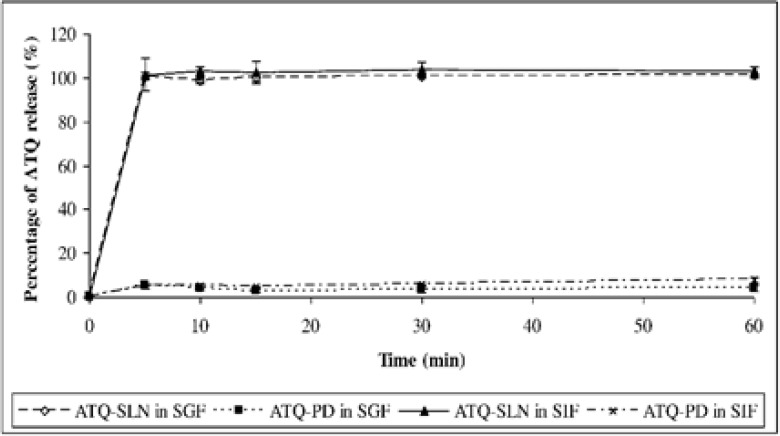
Dissolution of pure atovaquone (ATQ-PD) and atovaquone solid lipid nanoparticles (ATQ-SLN) in simulated gastric fluid (SGF) and simulated intestinal fluid (SIF), *n *= 3.

The release study showed an immediate and complete release of ATQ from the SLN formulation within 5 minutes which were 100.9 ± 0.7% in the SGF and 102.8 ± 2.4% in the SIF. The solubility of the native drug was observed at 3.8 ± 1.1 % in the SGF and 7.7 ± 1.3 % in the SIF after 60 minutes (Figure 4). An improvement in the dissolution of ATQ when formulated in the SLN was seen in both release media when compared to the same amount of the native drug (non-formulated ATQ). The dissolution of ATQ in the SLN formulation was improved as much as 97.1% in the SGF and 95.1% in the SIF. 

## Discussion

The deduced equations in Table 3 can be referred to understand the magnitude of single factors and interaction between factors in every design. The data was analysed by the software and only significant factors were included in the equations. A positive value indicates an increase in the response due to an increase in the magnitude of the factors and vice versa. For the ease of discussion, the impact of a single factor on both responses was simplified in Table 4. The negative impact of homogenizing cycle (factor A) on particle size can be explained by the high and prolonged energy input which helps to break down the droplets into nano-range particles. Prolongation of homogenization also improved the dispersion quality of the preparation in tripalmitin-and trilaurin-SLN.

**Table 4:** Impact of every factor on particle size and PdI of formulations in each design.

**Design**	**Particle size**		**PdI**	
	**Positive impact** a	**Negative impact** b	**Positive impact** a	**Negative impact** b
1	*C	*A, *B, *D	*C, *D	*B, A
2	*C, *D	*A, B	*C, *D	*A, *B
3	B, *C	*A, D	*C, *D	*A, *B
4	B, *C, D	*A	*C, D	A, B*
5	*C, *D	*A, *B	*A, *C, *D	B*
6	B, D	*A, C	*A, *C, D	B*

* *p* < 0.05

a increasing effect of the factors towards the dependent variables.

b decreasing effect of the factors towards the dependent variables.

A combination of surfactants usually produces smaller particle sizes due to the surface-active property of the surfactants. In the preliminary study, the use of Phospholipon 90H or the co-surfactant alone was proven insufficient as the layer of ATQ-dispersed lipid melt stuck to the container’s wall and was unable to be completely homogenized. When a combination of Phospholipon 90H and a co-surfactant was used, a complete homogenization could be achieved. Phospholipon 90H was kept as the main surfactant for every design because other than exhibiting emulsifying properties, it could help in the crystallization of the melted lipid. Bunjes and Koch (2005) reported that saturated phospholipids are able to increase the crystallization temperature (T_c_) of triglycerides by acting as a template for surface heterogenous nucleation (17). Theoretically, Phospholipon 90H (hydrogenated soy phosphatidylcholine) that was incorporated in the lipid melt will first bind to the vesicles and had limited mobility. This caused the emulsifier to be unable to sufficiently cover the new interfaces during crystallization and caused particle aggregation (18). Since a decrease in particle size is also associated with a profound increase in surface area, which will be more critical during homogenization where the increase in surface area can occur rapidly, a large amount of the emulsifier (*i.e*. co-surfactant) in the primary dispersion will help to cover the new surfaces (19). Therefore, a combination of Phospholipon 90H (Factor D) in the lipid phase and co-surfactants (Factor C; Tween 80 and Poloxamer 188) in the aqueous phase can help to produce nanosize dispersions. 

In Table 5, the SLN formulations that utilized Compritol 888 ATO as the lipid system showed low particle size and good PdI (design 5 and 6). The production of low particle sizes might be explained by the structural arrangement of the lipid. As reported, lipids with irregular orders such as Witepsol^®^ W35, which contains shorter mono- and diglyceride fatty acid chains, possess surface active properties (19) and helps in producing smaller particle size. This might also be the same case in Compritol 888 ATO as it also contains mono-, di- and triglycerides when compared to trilaurin and tripalmitin which solely contain triglycerides. However, the yield and entrapment efficiency of the formulations revealed an incompatibility of Compritol 888 ATO as the lipid matrix for the production of ATQ-SLN. Theoretically, the mixture of mono-, di-, and triglycerides in Compritol 888 ATO will cause irregular arrangements of the lipid and thus increase the accommodation of the drug during SLN production as previously reported (20, 21). However, the finding in this study clearly indicates that this is not necessarily the case. Compritol 888 ATO showed the least percentage of entrapment efficiency with less than 15% for all selected formulations. This might be due to the low solubility of ATQ in Compritol 888 ATO as a high encapsulation also depends on the sufficient solubility of the drug in the lipid melt (22). In contrast, higher entrapment efficiency was obtained from trilaurin-SLN. The relatively high entrapment when compared to tripalmitin-SLN can be attributed to the shorter chain length of the fatty acid in trilaurin. The same pattern has been observed in a study by Yassin *et al*. (2012) where the trimyristin-SLN (C14) showed superior characteristics when compared to the SLN produced using tristearin (C18) with the same composition. The study reported that trimyristin produced SLN formulations with smaller size, better quality of dispersion and higher entrapment efficiency (23).

**Table 5:** Yield, drug loading and encapsulation efficiency of selected formulations. Mean ± SD, *n* = 3

**Design**	**Code** a	**A**	**B**	**C**	**D**	**Zave**	**PdI**	**% Yield**	**% EE**
1	TPP 3	-	+	-	-	373.1 ± 25.5	0.438 ± 0.037	89.9 ± 1.0	33.5 ± 1.1
	TPP 4	+	+	-	-	271.4 ± 13.5	0.505 ± 0.062	90.3 ± 3.5	30.0 ± 0.9
2	TPT 4	+	+	-	-	188.4 ± 2.6	0.459 ± 0.010	92.5 ± 3.0	14.9 ± 0.8
	TPT 8	+	+	+	-	126.7 ± 1.4	0.504 ± 0.014	95.8 ± 1.8	38.9 ± 1.9
3	TLP 1	-	-	-	-	191.7 ± 5.0	0.416 ± 0.010	93.7 ± 1.3	11.6 ± 0.7
	TLP 4	+	+	-	-	192.5 ± 2.6	0.406 ± 0.034	92.0 ± 2.2	36.9 ± 0.7
	TLP 12	+	+	-	+	167.9 ± 6.0	0.465 ± 0.039	87.9 ± 3.8	37.1 ± 1.6
4	TLT 1	-	-	-	-	149.5 ± 3.9	0.434 ± 0.008	91.8 ± 1.2	15.1 ± 0.6
	TLT 3	-	+	-	-	213.6 ± 9.7	0.394 ± 0.018	89.7 ± 4.2	37.6 ± 1.8
	TLT 4	+	+	-	-	150.3 ± 7.5	0.268 ± 0.021	92.4 ± 2.2	35.3 ± 0.3
	TLT 8	+	+	+	-	103.8 ± 2.1	0.358 ± 0.011	93.5 ± 0.6	40.9 ± 0.7
	TLT 9	-	-	-	+	144.3 ± 1.7	0.495 ± 0.021	95.3 ± 1.4	18.7 ± 2.0
	TLT 11	-	+	-	+	165.8 ± 2.9	0.429 ± 0.019	95.9 ± 1.1	27.9 ± 2.0
	TLT 12	+	+	-	+	135.2 ± 23.4	0.502 ± 0.052	94.9 ± 2.5	41.9 ± 1.8
	* TLT 16	**+**	**+**	**+**	**+**	**95.3 **±** 0.9**	**0.425 **±** 0.008**	**95.5 **±** 1.0**	**45.7 **±** 1.8**
5	TBP 1	-	-	-	-	226.2 ± 21.7	0.268 ± 0.063	69.0 ± 11.2	4.6 ± 1.5
	TBP 2	+	-	-	-	229.8 ± 15.6	0.427 ± 0.068	74.5 ± 0.6	4.0 ± 0.5
	TBP 3	-	+	-	-	240.8 ± 6.9	0.219 ± 0.078	88.3 ± 1.7	5.7 ± 1.5
	TBP 4	+	+	-	-	222.3 ± 7.8	0.286 ± 0.067	88.8 ± 3.0	7.3 ± 1.9
	TBP 6	+	-	+	-	273.1 ± 20.3	0.491 ± 0.126	81.5 ± 2.2	5.5 ± 2.4
	TBP 7	-	+	+	-	256.6 ± 11.0	0.395 ± 0.135	90.4 ± 0.8	10.6 ± 3.0
	TBP 8	+	+	+	-	184.5 ± 18.8	0.369 ± 0.049	92.2 ± 2.1	7.2 ± 1.4
	TBP 9	-	-	-	+	260.1 ± 21.3	0.464 ± 0.043	80.2 ± 4.1	3.9 ± 0.6
	TBP 11	-	+	-	+	242.0 ± 5.3	0.360 ± 0.015	59.0 ± 12.8	11.7 ± 1.4
	TBP 12	+	+	-	+	202.3 ± 4.9	0.430 ± 0.050	78.4 ± 2.10	6.0 ± 0.8
	TBP 15	-	+	+	+	288.1 ± 6.7	0.342 ± 0.128	70.5 ± 4.2	6.8 ± 2.0
	TBP 16	+	+	+	+	256.1 ± 14.6	0.421 ± 0.116	78.0 ± 4.0	7.2 ± 2.0
6	TBT 3	-	-	-	-	263.9 ± 16.7	0.301 ± 0.046	88.3 ± 3.6	8.7 ± 2.3
	TBT 4	+	+	-	-	168.6 ± 2.2	0.360 ± 0.155	90.9 ± 3.2	13.9 ± 2.0
	TBT 7	-	+	+	-	208.6 ± 2.1	0.367 ± 0.043	91.2 ± 3.7	10.2 ± 3.4
	TBT 12	+	+	-	+	207.6 ± 20.7	0.386 ± 0.081	81.5 ± 3.1	13.1 ± 1.4

a Formulation codes according to the materials used in the preparation; TPP: tripalmitin, Phospholipon 90H and poloxamer 188; TPT: tripalmitin, Phospolipon 90H and Tween 80; TLP: trilaurin, Phospholipon 90H and poloxamer 188; TLT: trilaurin, Phospolipon 90H and Tween 80; TBP: Compritol 888 ATO, Phospholipon 90H and poloxamer 188; TBT: Compritol 888 ATO, Phospolipon 90H and Tween 80.

 Formulation TLT 16 was selected as the best formulation and further studied to improve the entrapment efficiency. The particle size of formulation TLT 16 significantly decreased (p < 0.05) while the entrapment efficiency significantly increased when the amount of ATQ in the formulation was reduced from 10 to 3 mg. It is known that reducing the amount of the drug will increase available spaces for the drug incorporation in the lipid (24). 

The characterization using DSC showed an alteration in the melting temperature of the lipid in the SLN. The shifting to a lower temperature can be explained by the smaller size of the particles and formation of less-ordered crystals of lipid in the SLN (25). Therefore, less energy was required to melt the substance and thus caused the reduction in melting temperature. Nonetheless, the peak of ATQ could not be seen in the thermogram of ATQ-SLN. This might be due to the encapsulation or amorphous state of the drug in the SLN as described by previous authors (15, 18). However, this could not be concluded with certainty as the peak of ATQ was also not seen in the physical mixture. Thus, the disappearance of the peak might also be caused by the small amount of the drug when compared to the excipients.

The release study showed a burst release of ATQ from the SLN as reported previously for other drugs (20, 26). This type of release could be explained by the large surface area due to the spherical shape and nanosize of the particles. It could also be caused by the enrichment of the drug on the outer layer and its deposition on the surface of the SLN. A drug-enriched outer layer of the SLN might have formed after the hot homogenizing process where an initial solidification of the lipid produced a central core containing the pure matrix. This eventually led to the encapsulation of the lipid core with a solid drug solution. The drug-enriched outer layer SLN provides a shorter diffusion path of the drug and causes the burst release (20). The surfactant in the formulation also helps in accelerating the solubilisation and release of ATQ from the SLN.

Despite the burst release, an improvement in the solubility of ATQ when compared to the pure drug would help to overcome the low aqueous solubility of ATQ which limits its bioavailability. The improvement could be associated with the large surface area of the nanoparticles and the presence of surfactants in the formulation. On the other hand, pure ATQ is a highly hydrophobic compound with a high surface resistance hindering the solubilisation of the free drug even in the presence of poloxamer 188 (1% w/w) in the release medium.

## Conclusion

An atovaquone-loaded SLN was successfully optimized using six sets of 2^4^ full factorial designs utilizing three types of glycerides and three types of surfactants. The analysis revealed that the optimum combination of surfactant system yielded SLN formulations with smaller particle sizes and a better quality of dispersion. The final optimized formulation (TLT 16) containing trilaurin as the lipid matrix was chosen and was further optimized and characterized. There was an improvement in the solubility despite the burst release. Hence, the formulated SLN could be an alternative solution to improve the solubility of ATQ which would eventually lead to an increase in the bioavailability of the drug. 
